# The CML experience to elucidate the role of innate T-cells as effectors in the control of residual cancer cells and as potential targets for cancer therapy

**DOI:** 10.3389/fimmu.2024.1473139

**Published:** 2024-11-15

**Authors:** Amandine Decroos, Sarah Meddour, Marine Demoy, Nathalie Piccirilli, Philippe Rousselot, Franck E. Nicolini, Stéphanie Ragot, Jean-Marc Gombert, André Herbelin, Alice Barbarin, Emilie Cayssials

**Affiliations:** ^1^ Université de Poitiers, Institut National de la Santé Et de la Recherche Médicale, Ischemie Reperfusion Métabolisme et Inflammation Stérile en Transplantation U1313, Poitiers, France; ^2^ Centre Hospitalier Universitaire de Poitiers, Délégation à la Recherche Clinique et à l'Innovation, Poitiers, France; ^3^ Centre Hospitalier de Niort, Laboratoire de Biologie Médicale Secteur Hématologie, Niort, France; ^4^ Hematology Department, Centre Hospitalier de Versailles, Versailles, France; ^5^ Institut National de la Santé Et de la Recherche Médicale U1184, Commissariat à l'Energie Atomique, Université Paris Saclay, Paris, France; ^6^ France intergroupe des Leucémies Myéloïdes Chroniques (FI-LMC), Centre Léon Bérard, Lyon, France; ^7^ Hematology Department & Institut National de la Santé Et de la Recherche Médicale 1052 Centre de Recherche en Cancérologie de Lyon, Centre Léon Bérard, Lyon, France; ^8^ Institut National de la Santé Et de la Recherche Médicale Centre d'Investigation Clinique 1402, Université de Poitiers, Poitiers, France; ^9^ Hematology Department, Centre Hospitalier Universitaire de Poitiers, Poitiers, France

**Keywords:** innate immunity, innate CD8 T-cells, T-cell effectors, perforin, PD-1, chronic myeloid leukemia (CML), biomarkers, predictive immune signature

## Abstract

Considering the general view that unconventional immune effectors play a major role in antitumor immunity, we recently postulated that the distinct new innate CD8 T-cell pool (co-expressing the transcription factor Eomesodermin and innate markers such as KIR/NKG2A) may counteract tumor cells, and thereby be potential target for cancer therapy. Here, to test this assumption, we used successfully targeted anti-leukemic therapy discontinuation (TFR) in chronic myeloid leukemia (CML). Numerical and functional status of innate CD8 T-cells, iNKT cells and γδ T-cells, in comparison with NK cells, was compared longitudinally between non-relapsed patients (i.e., with > 12 months TFR) and relapsed patients (i.e., who experienced molecular recurrence during the first 12 months after TKI cessation) in a prospective pilot cohort (n=32), starting from treatment discontinuation (D0). Perforin, a key cytotoxic immune player, was expressed in a significantly higher proportion of both innate CD8 T-cell and NK-cell subsets in non-relapsed patients, compared with relapsed patients at D0. In parallel, we assessed the expression of PD-1, an exhaustion marker used as target in cancer therapy. For all T-cell subsets, surface-expression level of PD-1 decreased in non-relapsed patients compared with relapsed patients at D0. This was particularly the case when considering iNKT cells for which surface-expression level of PD-1 even decreased relative to healthy control subjects. Lastly, we found a negative correlation between the proportion of innate CD8 T-cells expressing PD-1 and those expressing perforin in non-relapsed patients at D0. The fact that this was not the case in conventional CD8 T-cells is compatible with a reprogrammed effector profile preferentially targeting innate CD8 T-cells in non-relapsed patients. All in all, our results highlight NK cells and innate CD8 T-cells harboring cytotoxic content, as well as global downregulation of PD-1-expression on effector T-cells, as potential predictive functional signatures for successful TFR in CML. Considering innate CD8 T-cells, further investigations are needed to determine whether their possible contributory role in cancer surveillance in CML could be extended to other cancers, and also whether their targeting by immune cheek-point inhibitors could enhance their anti-tumoral functions.

## Introduction

Unconventional T-cells share functions with both adaptive and innate immune systems. Indeed, these cells are able on the one hand to produce INF-γ in response to the pro-inflammatory cytokines IL-12 and IL-18, and to exert cell-mediated cytotoxicity, which are innate functions, and on the other hand, to engage their T-cell receptor (TCR), which is an adaptive function. So, these cells do not fit into either adaptive or innate systems. In accordance with this notion, a recent study based on gene expression, metabolic profile and effector functions, described a continuous “innateness gradient” with adaptive cells at one end, followed by unconventional T cells, and natural cells (NK) cells at the other end (1).

Among unconventional T-cells, invariant natural killer T (iNKT) cells and γδ T-cells, representing 2 to 10% of circulating human T-cells, are well-recognized for their key role in immune surveillance against tumour cells (2–5). More recently, studies on mucosal-associated invariant T (MAIT) cells highlighted their relevant functions to tumor immunotherapy, including direct production of IFN-γ and cytotoxic molecules (6, 7). Alongside them, we recently identified a new contingent of unconventional T-cells, called innate CD8 T-cells, which can be distinguished from conventional CD8 T-cells by their combined expression of innate markers (panKIR/NKG2A) and the transcription factor Eomesodermin (Eomes). This unique phenotype is closely associated with IFN‐γ production in response to innate stimulation (8), corroborating with previous studies on memory CD8-T cells (9, 10). We have provided further evidence in favor of the innate character of this new unconventional T-cell compartment by documenting its position at an intermediate level in the innateness gradient in terms of innate IFN-γ production and decreased mitochondrial mass (11). All of these data support a role for innate CD8 T-cells in cancer immune surveillance (10, 12) and thereby as potential targets for cancer therapy.

To investigate the potential role of innate CD8 T-cells in the control of cancer, we have chosen a well-known hematological malignancy, chronic myeloid leukemia (CML). The development of CML follows a single chromosomal translocation, as demonstrated by experiments in mice, which showed that expression of the chimeric BCR-ABL protein was necessary and sufficient to trigger a myeloproliferative syndrome (13). We decided to analyze CD8 T-cells in patients in whom targeted anti-leukemic therapy discontinuation was successful despite the persistence of leukemic cells. We assume this clinical situation as a model for studying immune surveillance of tumors in humans based on several reports showing a close association between numerical increase in NK cells, which are well-known as major actors in anti-tumoral responses, and treatment-free remission (or TFR) (14–16).

Here, by considering a prospective pilot cohort of treatment withdrawal, we compared non-relapsed and relapsed CML patients during the first year following treatment cessation, in a comprehensive study of innate CD8 T-cells, along with iNKT and γδ T-cells, and NK cells, as a reference for innate immune signature of TFR, in terms of relative frequency and functional profile. We studied cytotoxic arsenal through perforin expression, so as to assess their involvement in residual disease control, and, on the other hand PD-1 expression in order to determine whether these cells may be targeted with immune checkpoint immunotherapy.

## Materials and methods

### Subjects and samples

Frozen peripheral blood mononuclear cells (PBMC) from 32 patients were obtained from a prospective pilot cohort (cf. **Supplementary Material**, patient characteristics in Table). Patients at least 18 years old, diagnosed with CML in chronic phase (CML-CP), who had been treated with TKI (tyrosin kinase inhibitor) and being at least 2 years with DMR, were included by the Poitiers CHU Hematology and Cellular Therapy Service, except for 10 patients from the OPTIM DASATINIB clinical trial (NCT01916785) (17). Among 32 patients, 8 relapsed, and 24 did not relapse after 12 months of TKI cessation. The term “relapsed patients” refers to patients having experienced MR (molecular recurrence), defined by the loss of MMR (major molecular response) during the 12 months following TKI cessation (18). The term “non-relapsed” patients refers to those having maintained their MMR during the 12 months following TKI cessation, the objective being to focus on early relapses. Venous blood was collected before treatment discontinuation (D0), and 3, 6 and 12 months after treatment discontinuation. PBMCs were isolated by density gradient centrifugation (Lymphocyte Separation Medium, Eurobio Scientific, CMSMSL01-01), resuspended in 90% fetal calf serum with 10% DMSO, and cryopreserved in liquid nitrogen until use. Frozen PBMCs from healthy control donors were obtained from the Blood Transfusion service [Etablissement Français du Sang (EFS Lyon)]. The study protocol was approved by the ethics committee at Poitiers University Hospital (Ethics Committee Ouest III, Poitiers, France, ministerial declaration n° DC 2008-565) and the study was conducted in compliance with Good Clinical practice guidelines. All participants gave written informed consent before inclusion in the trial.

### Cell culture

PBMCs were cultured in RPMI 1640 medium supplemented with antibiotics (penicillin and streptomycin) and 10% heat-inactivated fetal bovine serum. For IL-12/IL-18 stimulation, PBMCs from CML patients and healthy control donors were seeded at 1.10^6^ cells/mL into 24-well plates with IL-12 and IL-18 (both at 20 ng/mL, R&D systems) for 48 hours. GolgiSTOP (BD Biosciences) was added for the least 4 hours of culture.

### Flow cytometry

Phenotypic analyses by flow cytometry were performed either *ex vivo* or after culture in the presence of IL-12 + IL-18. Expression of the different markers was assessed by multicolor flow cytometry from staining panels with appropriate combinations of the following antibodies: anti-TCR-αβ BV510 (clone: IP26, BioLegend), anti-CD8 PE-Cy7 (clone: RPA-T8, BD Pharmingen), anti-Eomes eFluor^®^ 660 (clone:WD1928, eBiosciences), anti-Eomes PerCP-eFluor^®^ 710 (clone:WD1928, eBiosciences), anti-CD45RA PE (clone: HI100, BioLegend), anti-CD45RA BV510 (clone: HI100, BioLegend), anti-CCR7 PerCP-Cy5.5 (clone: G043H7), anti-TCR-γδ PE-Cy7 (clone: B1), anti-TCR Vα24-Jα18 PE (clone: 6B11, BioLegend), anti-CD3 PerCP-Cy5.5 (clone: UCHT1, BioLegend), anti-CD56 BV510 (clone: HCD56, BioLegend), were used to identify our populations of interest. Anti-PD-1 APC (clone: EH12.2H7, BioLegend), anti-Ki67 BV421 (clone: Ki-67, BioLegend), anti-Perforin FITC (clone: δG9, BD Biosciences), anti-IFNγ Alexa Fluor^®^ 488 (clone: 4S.B3, BioLegend), anti-panKIR2D PE (clone: NKVFS1, Miltenyi Biotech), anti-KIR3DL1/KIR3DL2 (CD158e/k) PE (clone: 5.133, Miltenyi Biotech), and anti-NKG2A (CD159a) PE (clone: REA110, Miltenyi Biotech), were used as read-out data. KIR/NKG2A referred to staining with a mix of anti-KIR2D, anti-KIR3DL1/KIR3DL2, and anti-NKG2A antibodies. For nuclear and intracytoplasmic staining, cells were fixed and permeabilized with an anti-human FoxP3 staining kit (eBiosciences) before staining with intracellular antibodies. Dead cells were excluded using the Zombie NIR™ Fixable Viability Kit (BioLegend).

Data were acquired on a FACSVerse™ cytometer with FACSuite™ software (BD Biosciences), and analyzed with the FlowJo software version 10 (BD Biosciences). The gating strategy used to define populations of interest is given in **Supplementary Figure S1**. Samples that failed to meet quality control criteria were excluded from analysis.

### Statistical analysis

Statistical analysis was performed using GraphPad Prism software version 9.5 (GraphPad, Dotmatics). Statistical significance of differences in frequencies and mean values was analyzed by the Mann-Whitney non-parametric test. The Spearman correlation test was used to assess the association between expressions of PD-1 and perforin. Results were considered to be statistically significant when p < 0.05. P-values between 0.05 and 0.15 are shown on the histograms. P-values below 0.05 are indicated on figure legends for healthy control donor and patient comparisons. Kaplan–Meier curves were plotted to assess the probability of molecular recurrence in patients with low versus high levels of the cell subsets studied. Immunological biomarkers were categorized in high and low levels building receiver operating characteristics (ROC) curves and calculating Youden index to choose the best cut-off value. A Log-Rank test was used to compare the probability of molecular recurrence between patients with high and low levels of each biomarker.

## Results

To document the role of immune effectors in controlling residual leukemia cells at the origin of non-relapse (or TFR) versus relapse (or MR) after TKI cessation, we performed comprehensive phenotyping of lymphoid effectors among peripheral blood samples from 32 patients in our CML pilot cohort of TKI discontinuation and 12 healthy control donors. We aimed to compare the relative frequency and functional profile of NK cells, effector CD8 T-cells and non-conventional T-cell populations (for gating strategy, see **Supplementary Figure S1**), between relapsed and non-relapsed patients at the moment of TKI discontinuation.

### Relative frequencies of immune effector subsets did not significantly differ between relapsed and non-relapsed patients

**Figure 1:**
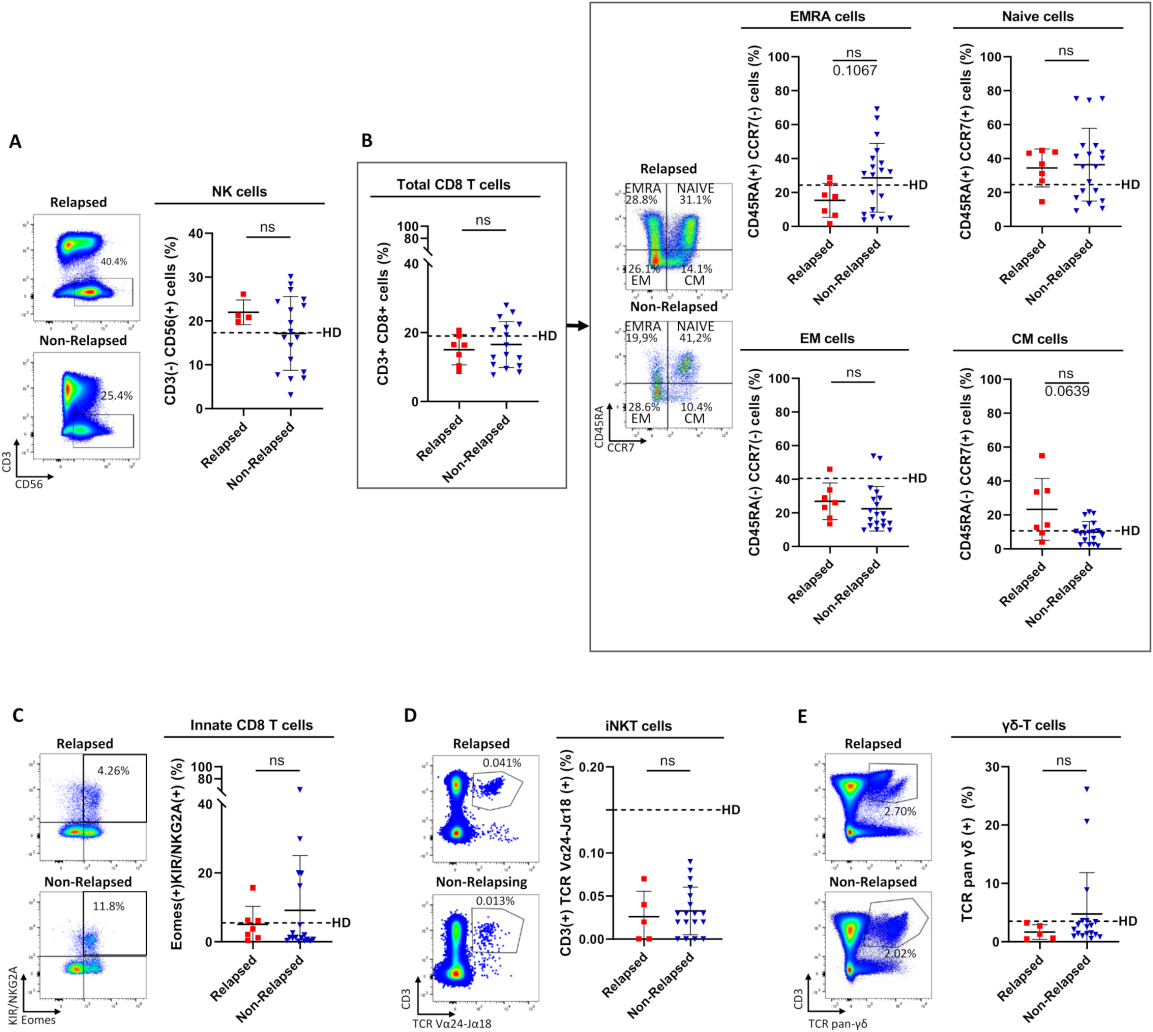
Comparison of relative frequencies of circulating immune effector subsets between relapsed and non-relapsed patients at the moment of TKI discontinuation. **(A)** Frequencies of NK cells, defined as CD3(-)CD56(+) cells, among live lymphocytes from relapsed (n=4) and non-relapsed (n=18) patients. Healthy control donors (HD) mean: 17.3% ± 8.1% (n=11). **(B)** Frequencies of total CD8 T cells among live lymphocytes (left panel) and of EMRA, Naive, EM, and CM cells based on the expression of CD45RA and/or CCR7 among total CD8 T-cells, defined as TCR-αβ(+)CD8(+) cells (right panel), from relapsed (n=7) and non-relapsed (n=19) patients. HD means: 19% ± 5.6% of CD8-T cells among live lymphocytes; 24.4% ± 5.4% of EMRA cells, 24.6% ± 18.6% of Naive cells, 10.6% ± 5.7% of CM cells, 40.5% ± 14.3% of EM cells among total CD8 T-cells (n=11). Of note, for EM cells, differences were significant between HD and non-relapsed patients (p-value: 0.002). **(C)** Frequencies of innate CD8-T cells, defined as TCR-αβ(+)CD8(+) Eomes(+)KIR/NKG2A(+), among total CD8 T-cells from relapsed (n=7) and non-relapsed (n=18) patients. HD mean: 5.5% ± 7.4% (n=13). **(D)** Frequencies of iNKT cells, defined as CD3(+)TCR-Vα24-Jα18(+), among live lymphocytes from relapsed (n=5) and non-relapsed (n=18) patients. HD mean: 0.15% ± 0.2% (n=11). **(E)** Frequencies of γδ T-cells, defined as CD3(+)TCR-pan-γδ(+), among live lymphocytes from relapsed (n=5) and non-relapsed (n=18) patients. HD mean: 3.5% ± 2.9% (n=11). Representative dot plots show relative frequencies of the different populations of interest in relapsed (top left) and non-relapsed (bottom left) patients. Histograms represent cohort analysis of relative population frequencies (mean ± SD). Each dot represents a relapsed (red squares) or a non-relapsed (blue triangles) patient. Dotted lines represent relative frequency mean for each population of interest in HD. Statistical significance was determined by the two-tailed Mann-Whitney non-parametric test, ns: not significant.

Relative proportion of NK (CD3(-)CD56(+)) cells did not significantly differ between non-relapsed and relapsed patients (**Figure 1A**). CD8 T (TCR-αβ(+)CD8(+)) cells are functionally divided into four subpopulations, according to cell surface expression of CCR7 and CD45RA: naive (CCR7(+), CD45RA(+)); central memory (CM, CCR7(+)CD45RA(+); effector memory (EM, CCR7(-)CD45RA(-)); and terminally differentiated effector memory (EMRA, CCR7(-)CD45RA(+)) (19). There was no significant difference between the two groups of patients in the percentages of total CD8 T-cells or the four CD8 T-cell subpopulations (**Figure 1B**).

As for NK cells and the four CD8 T-cell subpopulations, percentages of non-conventional effector T-cell populations, namely innate CD8 T (TCR-αβ (+)CD8(+))Eomes(+)KIR/NKG2A(+)) cells (**Figure 1C**), iNKT (CD3(+)TCR-Vα24-Jα18(+)) cells (**Figure 1D**), and γδ-T (CD3(+)TCR-γδ(+)) cells (**Figure 1E**), did not significantly differ between non-relapsed and relapsed patients.

### TFR was closely associated with higher frequencies of NK cells and innate CD8 T-cells expressing perforin.

**Figure 2:**
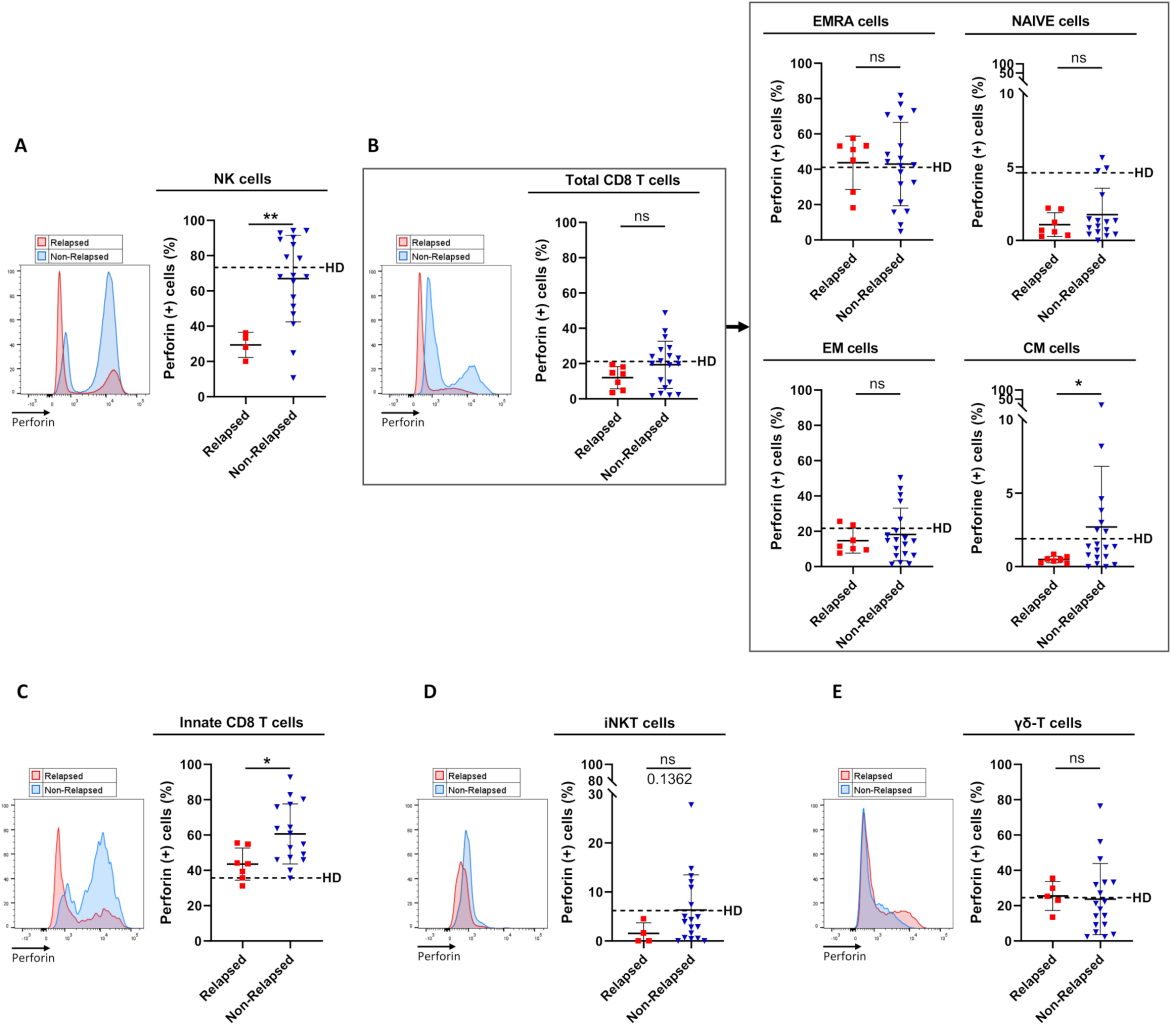
Frequencies of perforin-expressing cells among circulating immune effector subsets between relapsed and non-relapsed patients at the moment of TKI discontinuation. **(A)** Frequency of perforin-expressing cells among NK cells, defined as CD3(-)CD56(+) cells, from relapsed (n=4) and non-relapsed (n=18) patients. Healthy control donors (HD) mean: 73.3% ± 16.3% (n=11). Of note, differences were significant between HD and relapsed patients (p-value: 0.0015). **(B)** Frequency of perforin-expressing cells among total CD8 T-cells, defined as TCR-αβ(+)CD8(+) cells (left panel), and among EMRA, Naive, EM, and CM cells based on the expression of CD45RA and/or CCR7 (right panel) from relapsed (n=7) and non-relapsed (n=19) patients. HD means: 21.2% ± 20.6% of total CD8 T-cells; 41.1% ± 24.9% of EMRA cells; 4.6% ± 7.2% of Naive cells; 21.7% ± 19.4% of EM cells; 1.9% ± 2.5% of CM cells (n=11). Of note, for CM cells, differences were significant between HD and relapsed patients (p-value: 0.0441). **(C)** Frequency of perforin-expressing cells among innate CD8 T-cells, defined as TCR-αβ(+)CD8(+)Eomes(+)KIR/NKG2A(+), from relapsed (n=7) and non-relapsed (n=16) patients. HD mean: 35.7% ± 14.5% (n=10). Of note, differences were significant between HD and non-relapsed patients (p-value: 0.0015). **(D)** Frequency of perforin-expressing cells among iNKT cells, defined as CD3(+)TCR-Vα24-Jα18(+), from relapsed (n=4) and non-relapsed (n=18) patients. HD mean: 6.2% ± 10.3% (n=11). **(E)** Frequency of perforin-expressing cells among γδ T-cells, defined as CD3(+)TCR-pan-γδ(+), from relapsed (n=5) and non-relapsed (n=18) patients. HD mean: 24.5% ± 17.9% (n=11). Representative overlays flow cytometry histograms show perforin expression in the different populations of interest in relapsed (red line) and non-relapsed (blue line) patients. Histograms represent cohort analysis of relative population frequencies (mean ± SD). Each dot represents a relapsed (red squares) or a non-relapsed (blue triangles) patient. Dotted lines represent frequency mean of perforin-expressing cells for each population of interest in HD. Statistical significance was determined by the two-tailed Mann-Whitney non-parametric test, ns: not significant, *p < 0.05; **p < 0.01.

We next studied the functional status of circulating immune effector cells by analyzing *ex vivo* perforin expression (**Figure 2**), a key cytotoxic player known to be mainly expressed by NK cells and effector T-cell populations.

In both healthy control donors and non-relapsed patients, perforin was shown to be expressed by a large majority of NK cells (nearly 80% of positive cells), a proportion that dramatically decreased (only 30-35% of positive cells) in relapsed patients (**Figure 2A**). When considering T-cell compartments (**Figures 2B–D**), in healthy control donors, the propensity to express perforin was an expected common feature of effector T-cells, i.e. EMRA, EM, and innate CD8 T-cell subpopulations (**Figures 2B, C**), iNKT cells (**Figure 2D**), and γδ-T cells (**Figure 2E**), with a proportion of perforin-expressing cells ranging from 7 to 40%, compared with less than 2% for the other T-cell subsets (naive and CM CD8 T-cells). In non-relapsed patients, among all effector T-cell subsets, only the innate CD8 T-cell pool contained a significantly higher proportion of perforin-positive cells, compared with relapsed patients. Remarkably, the proportion of perforin-positive innate CD8 T-cells was even higher in non-relapsed patients than in healthy control donors (p-value: 0.0015). Another effector activity of innate CD8 T-cells is their ability to produce IFN-γ in response to innate stimulation by IL-12/IL-18. However, this NK-like activity of innate CD8 T-cells did not appear to significantly differ between non-relapsed and relapsed patients, with IFN-γ levels similar to those found in healthy control donors (**Supplementary Figure S2**).

**Figure 3:**
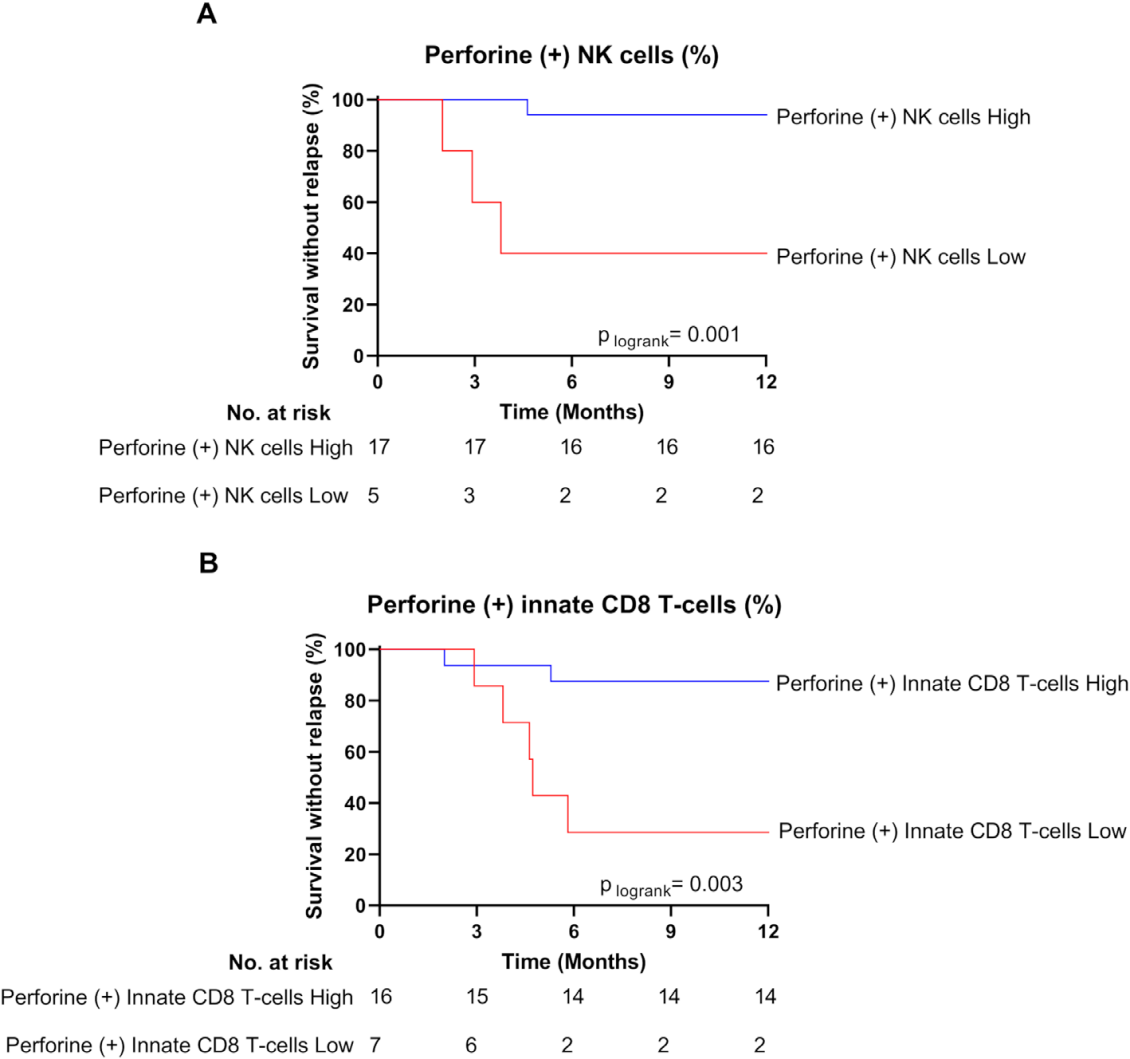
High frequencies of perforin-expressing NK cells and innate CD8 T-cells are associated with treatment-free remission. **(A)** Survival without relapse over the first 12 months following TKI cessation in patients with high (blue line) and low (red line) frequency of perforin-expressing NK cells at the moment of TKI cessation. The optimal cut-off value of frequency of perforin-expressing NK cells (38.75%) was calculated using the Youden index. **(B)** Survival without relapse over the first 12 months following TKI cessation in patients with high (blue line) and low (red line) frequency of perforin-expressing innate CD8 T-cells at the moment of TKI cessation. The optimal cut-off value of frequency of perforin-expressing innate CD8 T-cells (45%) was calculated using the Youden index. The number of subjects at risk is shown below the curves. Statistical significance was determined by Log-Rank test.

Finally, the higher perforin expression frequency evidenced at the moment of TKI cessation in NK cell and innate CD8 T-cell subsets from non-relapsed patients appeared relatively robust. Indeed, for both cell subsets, when patients were dichotomized to low- and high- perforin expressing-cell-frequency groups according to ROC curves and the Youden index, the difference in molecular relapse-free survival at 6 months between the two groups was significant (87.5% vs 28.5% (p=0.001) and 94.1% vs 40.0% (p=0.003) for NK cell and innate CD8 T-cell frequencies, respectively) (**Figure 3**).

Furthermore, over time (3, 6, and 12 months) after TKI cessation, the frequency of cells expressing perforin remained significantly higher among the NK cell subset (p-values: 0.0264, 0.0048, 0.0645, respectively), and tended to remain higher among the innate T-cell subset (p-values: 0.2523, 0.1409, 0.1353, respectively), compared with relapsed patients at the moment of TKI cessation (**Supplementary Figure S3**).

In our cohort, the majority of non-relapsed patients were treated by dasatinib, which is known for targeting both NK cells and effector T-cells (20–24). As shown in **Supplementary Figure S4**, frequencies of perforin-expressing innate CD8 T-cells and NK cells did not differ with regard to the type of TKI received by the non-relapsed patients, thereby ruling out a selective effect on perforin expression.

All in all, these data lead us to consider the increased proportion of perforin-expressing NK cells and innate CD8 T-cells as functional signatures of TFR.

### TFR was closely associated with decreased PD-1 expression on both effector CD8 T-cells and non-conventional T-cells

**Figure 4:**
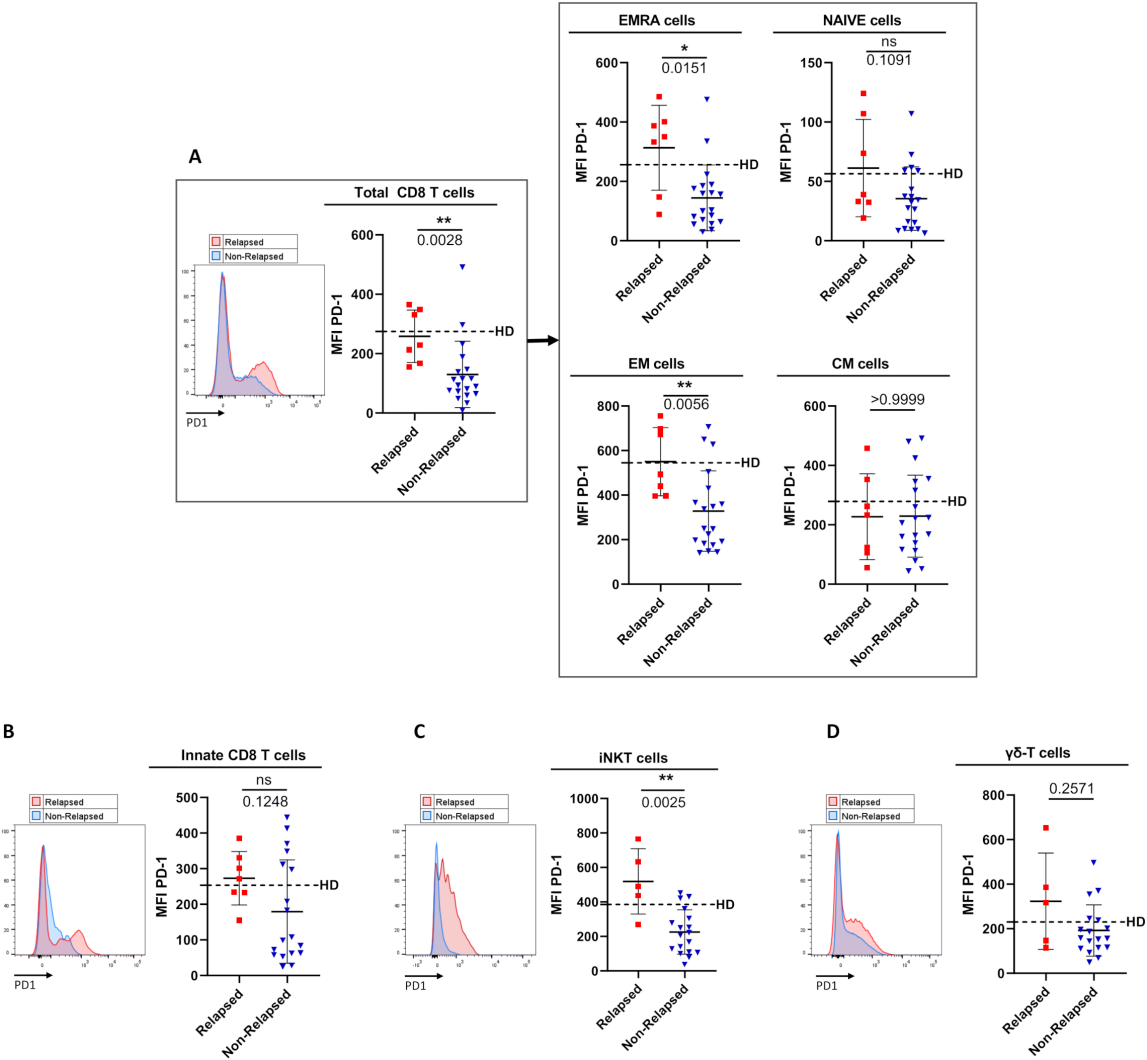
Comparison of PD1 surface-expression level on circulating immune effector T-cell subsets between relapsed and non-relapsed patients at the moment of TKI discontinuation. **(A)** PD-1 cell-surface MFI (mean fluorescence intensity) levels among total CD8 T-cells, defined as TCR-αβ(+)CD8(+) cells (left panel), and among EMRA cells, Naive cells, EM cells, and CM cells based on the expression of CD45RA and/or CCR7 (right panel) from relapsed (n=7) and non-relapsed (n=19) patients. Healthy control donors (HD) means: 274.6 ± 168.8 of total CD8 T-cells; 256.1 ± 168.8 of EMRA cells; 56.4 ± 40.2 of Naive cells; 545.3 ± 201.5 of EM cells; 278.7 ± 139 of CM cells (n=11). Of note, differences were significant between HD and non-relapsed patients for total CD8-T cells, EMRA cells and EM cells (p-values: 0.0031; 0.0164; 0.0037 respectively). **(B)** PD-1 cell-surface MFI on innate CD8 T-cells, defined as TCR-αβ(+)CD8(+)Eomes(+)KIR/NKG2A(+), from relapsed (n=7) and non-relapsed (n=16) patients. HD mean: 253.4 ± 125.1 (n=10). **(C)** PD-1 cell-surface MFI on iNKT cells, defined as CD3(+)TCR-Vα24-Jα18(+), from relapsed (n=5) and non-relapsed (n=11) patients. HD mean: 385.1 ± 113 (n=18). Of note, differences were significant between HD and relapsed patients (p-value: 0.0051). **(D)** PD-1 cell-surface MFI on γδ T-cells, defined as CD3(+)TCR-pan-γδ(+), from relapsed (n=5) and non-relapsed (n=17) patients. HD mean: 230.1 ± 93.2 (n=11). Representative overlays flow cytometry histograms show PD-1 expression in the different populations of interest in relapsed (red line) and non-relapsed (blue line) patients. Histograms represent cohort analysis of PD-1 cell-surface MFI (mean ± SD). Each dot represents a relapsed (red squares) or a non-relapsed (blue triangles) patient. Dotted lines represent frequency mean of PD-1 cell-surface MFI for each population of interest in HD. Statistical significance was determined by the two-tailed Mann-Whitney non-parametric test, ns: not significant, *p < 0.05; **p < 0.01.

We next assessed the expression of PD-1, an activation marker also routinely used to evaluate T-cell exhaustion during chronic pathological situations, including cancers (25) (**Figure 4**).

**Figure 5:**
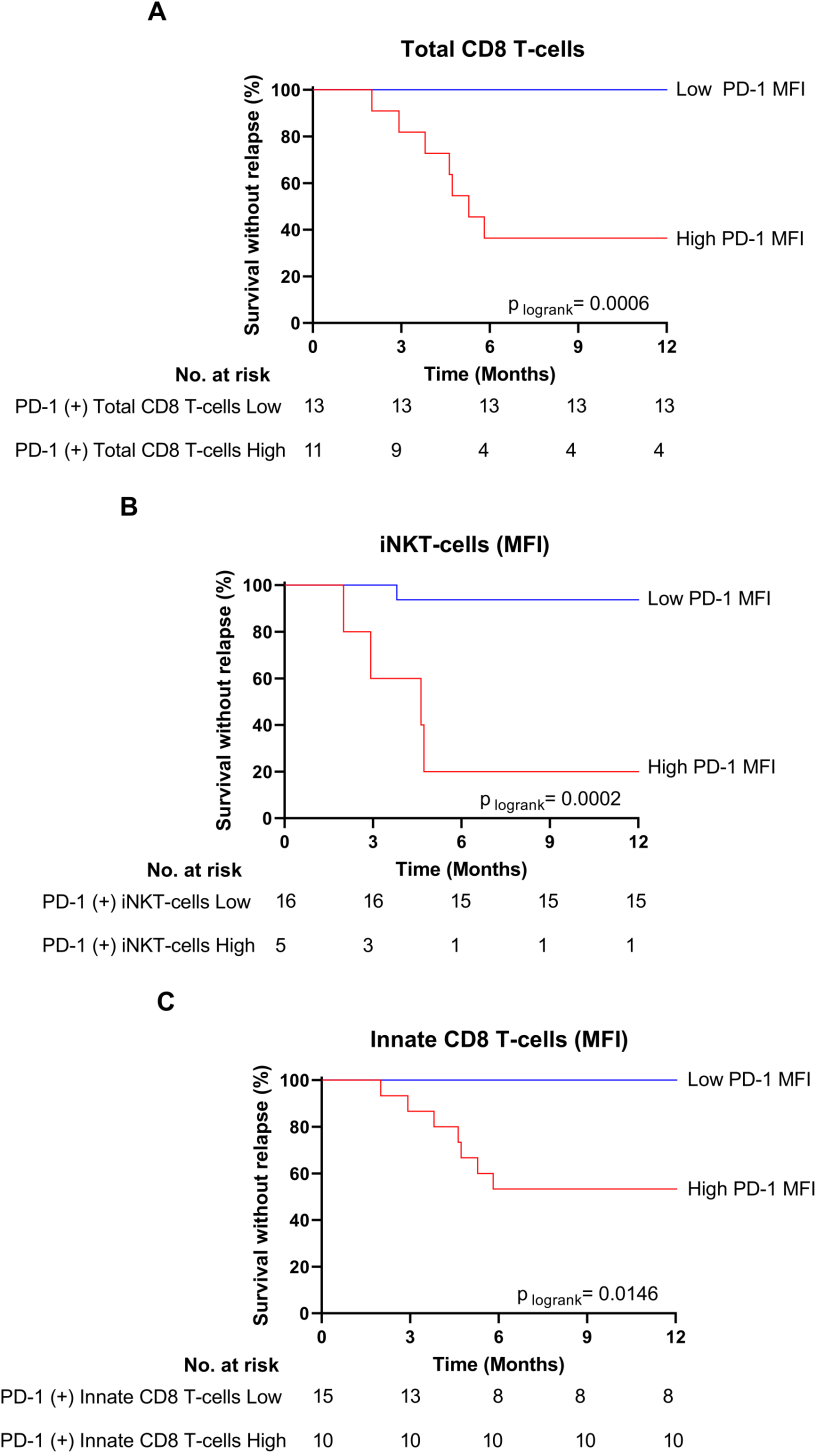
Low PD-1 MFI on total CD8 T-cells, iNKT innate CD8 T-cells are associated with treatment-free remission. **(A)** Survival without relapse over the first 12 months following TKI cessation in patients with low (blue line) and high (red line) PD-1 MFI on total CD8 T-cells at the moment of TKI cessation. The optimal cut-off value of PD-1 MFI on total CD8 T-cells (151) was calculated using the Youden index. **(B)** Survival without relapse over the first 12 months following TKI cessation in patients with low (blue line) and high (red line) PD-1 MFI on iNKT cells at the moment of TKI cessation. The optimal cut-off value of PD-1 MFI on iNKT cells (433.5) was calculated using the Youden index. **(C)** Survival without relapse over the first 12 months following TKI cessation in patients with low (blue line) and high (red line) PD-1 MFI on innate CD8 T-cells at the moment of TKI cessation. The optimal cut-off value of PD-1 MFI on total CD8 T-cells (131.5) was calculated using the Youden index. The number of subjects at risk is shown below the curves. Statistical significance was determined by Log-Rank test.

In relapsed patients, when compared to healthy control donors, PD-1 appeared increased, but not significantly, in terms of cell-surface expression level on several effector T-cell subsets, namely EMRA CD8 T-cells, iNKT cells and γδ T-cells. In clear contrast, as compared to the same healthy control group, non-relapsed patients showed a dramatic reduction in PD-1 expression level on EMRA CD8 T-cells (p-value: 0.017), EM CD8 T-cells (p-value: 0.004), and iNKT cells (p-value: 0.0005), providing evidence that down-regulation of PD-1 is systemic in effector T-cells from this group of patients. As a result, cell-surface MFI level of PD-1-expressing cells among EMRA CD8 T-cells (p-value: 0.015), EM CD8 T-cells (p-value: 0.006), and iNKT cells (p-value: 0.0025) significantly decreased in non-relapsed patients compared to their relapsed counterparts. Moreover, for total CD8 T-cells, and iNKT cells, when patients were dichotomized to low- and high- PD-1 MFI groups according to ROC curves and the Youden index, the difference in molecular relapse-free survival at 6 months between the two groups was significant (100.0% vs 36.3% (p=0.0006), and 94.1% vs 20.0% (p=0.0002) for CD8 T-cell, and iNKT-cell PD-1 MFI, respectively) (**Figures 5A, B**). Furthermore, this phenomenon was maintained over time (3-to-12 months) after discontinuation of TKI therapy (see legend of **Supplementary Figure S5**).

In innate CD8 T-cells, a decrease in PD-1 surface-expression level was also observed in non-relapsed patients compared to their relapsed counterparts, albeit less marked than for other effector CD8 T-cell subpopulations. This conclusion applied both at the moment of TKI discontinuation (p-value: 0.026) and over the following months (p-values for 6 and 12 months: 0.0813 and 0.0392, respectively) (**Supplementary Figure S5**). However, when patients were dichotomized to low- and high- PD-1 MFI groups, on innate CD8 T-cells, according to ROC curves and the Youden index, the difference in molecular relapse-free survival at 6 months between the two groups was significant (100% vs 53,3% (p=0.0146) (**Figure 5C**).

Lastly, as for perforin, type of TKI, which was used as a treatment prior to its discontinuation, was found to have no impact on PD-1 expression level in immune effector cells from non-relapsed patients (**Supplementary Figure S6**).

### Negative correlation between PD-1- and perforin- expression by innate CD8 T-cells

**Figure 6:**
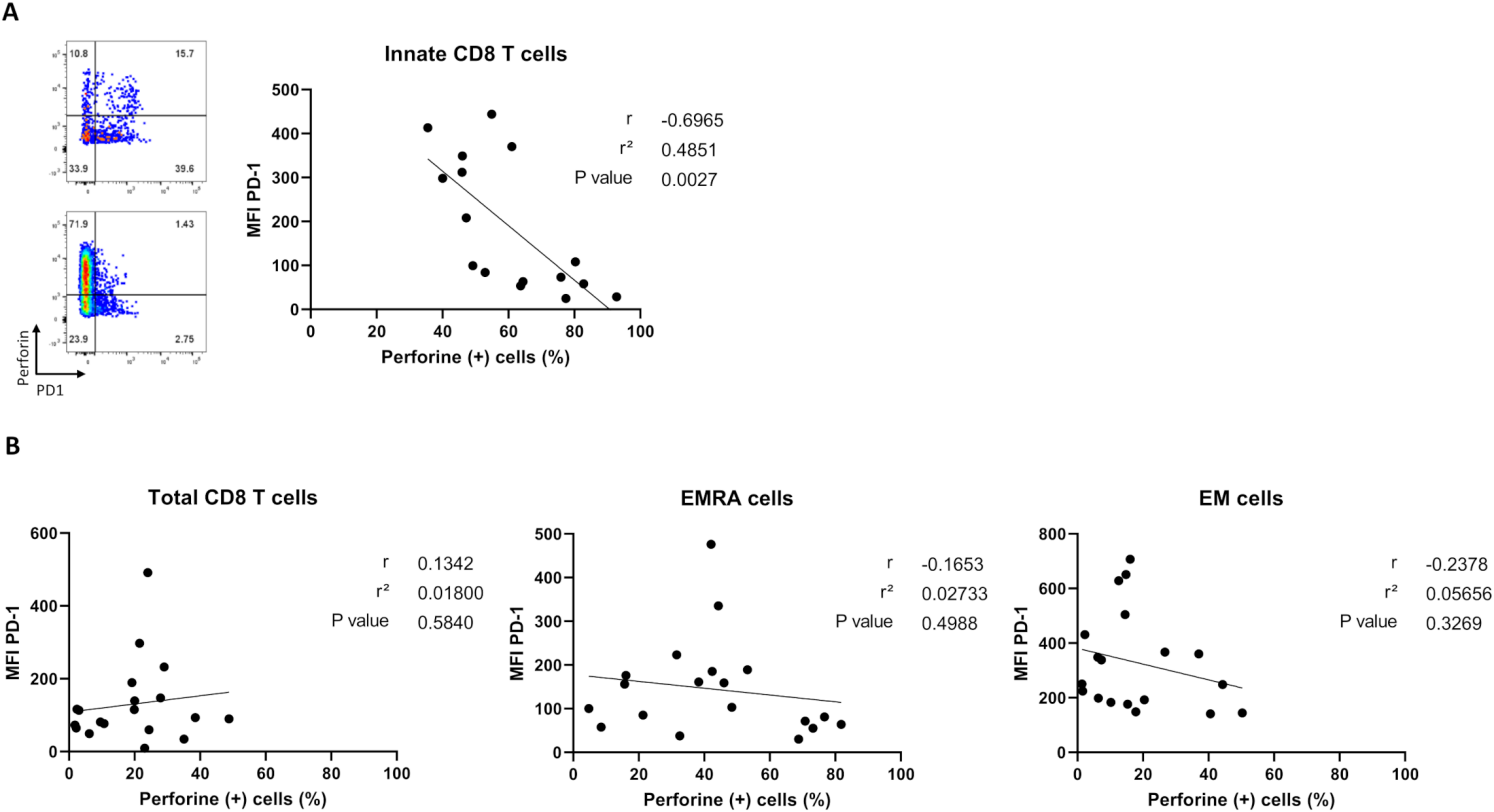
Correlation analysis between percentages of perforin-expressing cells and PD-1 cell-surface MFI levels among CD8 T-cell subpopulations in non-relapsed patients at the moment of TKI discontinuation. **(A)** Correlation plots showing negative correlation between PD-1 cell-surface MFI and perforin-expressing innate CD8-T cells, defined as TCR-αβ(+)CD8(+)Eomes(+)KIR/NKG2A(+), (n=16). Representative dot plots show frequencies of PD1 versus perforin-expressing innate CD8 T cells from two non-relapsed patients (left). **(B)** Correlation plots showing no correlation between PD-1 cell-surface MFI and percentages of perforin-expressing cells among total CD8-T cells, defined as TCR-αβ(+)CD8(+), EMRA cells, defined as TCR-αβ(+)CD8(+)CCR7(-)CD45RA(+),and EM cells, defined as TCR-αβ(+)CD8(+)CCR7(-)CD45RA(-), frequencies (n= 19). Spearman correlation test.

To test whether the higher proportion of perforin-expressing CD8 T-cells was associated with a lower exhaustion-like status in non-relapsed patients, we analyzed correlations between percentages of perforin-expressing cells and PD-1 cell-surface MFI levels among the three CD8 T-cell effector subpopulations at the time of TKI discontinuation (**Figure 6**). We found a significant negative correlation between perforin- and PD-1 expression by innate CD8 T-cells (**Figure 6A**), a phenomenon that was not found when considering the total CD8 T-cell pool or EMRA and EM CD8 T-cell effector subpopulations (**Figure 6B**).

## Discussion

Invariant natural killer T (iNKT) cells and γδ T-cell subsets are well-recognized for their key role in immune surveillance against tumor cells (2–5). For its part, the new innate (Eomes(+)panKIR/NKG2A(+)) CD8 T-cell contingent (8), due to its cytotoxic arsenal through perforin expression and IFN‐γ production in response to innate stimulation, could also play a role in the control of cancer (8, 12). Here, to test this hypothesis, we chose CML, a well-known haematological malignancy, in the context of residual disease control following TKI treatment discontinuation.

Using our comprehensive analysis at the moment of TKI discontinuation, we first documented that the proportion of perforin-expressing innate CD8 T-cells was higher in non-relapsed patients than in relapsed patients. Mechanistically, the fact that higher frequency of perforin-expressing but not IFN-γ-expressing innate CD8 T-cells was closely associated with TFR suggests that these cells directly target residual leukemic cells rather than exerting a bystander effect. However, we cannot rule out a co-action of IFN-γ facilitating successful TKI discontinuation. It will also be interesting to extend our analysis to the cytotoxic potential against leukemic cell lines.

We also studied possible control of the residual disease by other unconventional T-cells, but found no differences of perforin expression frequencies in iNKT and γδ T-cells between non-relapsed and relapsed patients. It would be interesting to extend our study to MAIT cells, because of their potential anti-tumor functions, and because only a few studies so far have focused specifically on this non-conventional T-cell subset in blood cancers (6, 7, 26).

Finally, as with innate CD8 T-cells, we found a higher proportion of perforin-expressing NK cells in non-relapsed than in relapsed patients. This finding, while being in concordance with the previously potential role attributed to NK cells in maintained TFR following TKI discontinuation, also underlined the possible existence of a coordinate role of the two innate cell compartments in residual disease control. Importantly, for both NK cells and innate CD8 T-cells, the higher proportion of cells expressing perforin was not dependent on the type of TKI used as treatment, suggesting that these cells may ensure immune surveillance in other haematological malignancies, and even solid cancers.

In a general view, we speculate that an “innateness score” taking into account all non-conventional T-cell subsets could represent a useful strategy for identifying novel innate signatures associated with effective treatment response against tumors. To this end, it will be interesting to evaluate whether the “innateness gradient”, based on gene expression, metabolic profile and effector functions, with adaptive T cells at one end, followed by unconventional T cells and NK cells at the other (1), may represent a useful means of “innateness” scoring. Moreover, analysis of innateness-associated transcription factors (PLZF, T-BET and Eomes) in combination with functional (granzyme B and K) and exhaustion (TIM-3 and TIGIT) markers may enable us to identify coordinated elements among unconventional T-cells that could contribute to global anti-tumoral response.

When applied to CML, an “innateness score” might help to identify coordinate elements involved in successful response to TKI treatment, given that unconventional T-cells are numerically and functionally impaired at diagnosis (8, 27–29), and show recovered frequencies and functions in patients responding to TKI therapy (12, 30–32). More generally, an “innateness score” might be used as a prognostic factor in other cancers such as multiple myeloma, where unconventional T-cells, i.e., iNKT cells, and γδ T-cells, are functionally impacted at multiple myeloma diagnosis (33, 34). Considering innate CD8 T-cells, our laboratory has observed a trend toward increased frequency in the bone marrow from patients at multiple myeloma diagnosis, suggesting their involvement in disease control (unpublished data).

In parallel, we studied PD-1 expression to assess whether unconventional T-cells, especially innate CD8-T cells, could be potential targets for cancer immunotherapy. PD-1 is involved in activation and exhaustion of T-cells (35), and its aberrant expression has been described in cancer immunity (25). Here, the fact that PD-1 expression levels were lower in non-relapsed than in relapsed patients and healthy control donors led us to speculate that PD-1 on effector T-cells from non-relapsed patients is a signature of reprogramming in these patients rather than a signature of exhaustion in relapsed patients.

We also highlighted a trend towards a lower level of PD-1 expression on innate CD8 T-cells in non-relapsed patients in comparison to relapsed patients and healthy donors. Moreover, we revealed a negative correlation between perforin and PD-1 expression on innate CD8 T-cells that was not found on other effector CD8 T-cell subpopulations, thereby attesting that this phenomenon is specific to innate CD8-T cells in patients having achieved TFR. It is known that *in vitro* PD-1 blockade improves cytotoxic functions, such as IFN-γ production, on unconventional T-cells, i.e. MAIT, iNKT and γδ T-cells (36–38). It would be interesting to study *in vitro* the impact of blocking the immune checkpoint PD-1 on innate CD8-T cells from healthy donors on their cytotoxic functions, and thereby attempt to reproduce the “PD-1 signature” associated with non-relapsed CML patients.

Interestingly, the lower expression level of PD-1 was widespread to iNKT cells and conventional CD8-T cells, such as EM, EMRA and naïve CD8 T-cells in non-relapsed patients, leading us to speculate on a possible benefit of immunotherapeutic strategies targeting PD-1, the objective being to enhance residual disease control. As for perforin expression, this phenomenon was independent of the type of TKI used as treatment, suggesting that our results may be applied to cancers in which TKI are not used for treatment.

At a clinical level, we can speculate on the possible benefit of TKI treatment in combination with immunotherapeutic strategies targeting PD-1 in order to achieve TFR in patients not exhibiting a decreased proportion of PD1-expressing T-cells. We also believe that an ‘innateness score’ could represent a useful strategy for identifying novel innate signatures associated with resolutive immune-mediated tumor therapy.

Taken together, by studying their involvement in control of residual disease in CML our results provide the first evidence of a contributory role of innate CD8 T-cells in cancer surveillance. Of course, our study is based on a prospective pilot cohort with a limited number of patients, and consequently needs to be confirmed in a larger prospective multicentric cohort. In a wider context, and considering that innate CD8 T-cells are present locally in solid tumors such as ovarian carcinosis (10), further investigations are needed to determine whether their possible contributory role in cancer surveillance in CML could be extended to other cancers, and also whether their targeting by immune cheek-point inhibitors, especially anti-PD1, could enhance their anti-tumoral functions.

## Data Availability

The raw data supporting the conclusions of this article will be made available by the authors, without undue reservation.
