# Supplementary material for: The CML experience to elucidate the role of innate T-cells as effectors in the control of residual cancer cells and as potential targets for cancer therapy
Source: Front Immunol. 2024 Nov 15;15:1473139. doi: 10.3389/fimmu.2024.1473139 (PMC11604645; doi:10.3389/fimmu.2024.1473139)
Supplement: Supplementary file 1 [file DataSheet1.pdf]

<u>Table</u>: Patient Characteristics

N°	Sex	Age at D0	Treatment	Treatment Duration (months)	Molecular response (log)	MR (months)	Cohort
**1**	F	68	Ima	43	5	4	MR
**2**	M	80	IFNα-Ima	145	4,5	5	MR
**3**	F	38	Hydrea-Ima	57	5	5	MR
**4**	F	55	Dasa	95	5	3	MR
**5**	M	43	Dasa	103	4,5	2	MR
**6**	M	43	Ima	62	4,5	6	MR
**7**	M	61	IFNα-Dasa	62	5	3	MR
**8**	F	37	Hydrea-Dasa	69	5	0	MR
**9**	F	29	Hydrea-Ima-IFNα-Dasa	168	4,5	NA	TFR
**10**	F	73	Hydrea-Actos-Ima	136	4,5	NA	TFR
**11**	M	82	Dasa	63	5	NA	TFR
**12**	M	57	Ima - Nilo	>82	4	NA	TFR
**13**	M	81	IFNα-Ima-Dasa	>217	4,5	NA	TFR
**14**	M	78	Dasa	102	5	NA	TFR
**15**	M	68	Ima - Nilo	122	5	NA	TFR
**16**	M	62	IFNα-Actos-Ima	>220	4	NA	TFR
**17**	M	59	Aracytine-Ima-Nilo-Dasa	166	5	NA	TFR
**18**	M	26	Ima	111	4,5	NA	TFR
**19**	M	54	IFNα-Ima	55	4,5	NA	TFR
**20**	M	65	Ima	183	5	NA	TFR
**21**	F	70	Nilo	83	4,5	NA	TFR
**22**	F	64	Ima - Dasa	191	4,5	NA	TFR
**23**	F	72	Ima	164	5	NA	TFR
**24**	F	53	Dasa	71	4,5	NA	TFR
**25**	F	53	Dasa	100	4	NA	TFR
**26**	M	66	Dasa	34	5	NA	TFR
**27**	M	40	Dasa	>54	5	NA	TFR
**28**	M	53	Dasa - Bosu	>64	5	NA	TFR
**29**	F	64	Dasa	72	5	NA	TFR
**30**	F	57	Dasa	44	5	NA	TFR
**31**	F	80	Dasa	76	5	NA	TFR
**32**	F	60	Dasa	75	5	NA	TFR

NA: not applicable
Ima: imatinib ; Dasa: dasatinib ; Nilo: nilotinib ; Bosu: bosutinib
MMR: major molecular remission
D0: treatment discontinuation
MR: molecular recurrence
TFR: treatment-free remission

Supplementary Figure 1

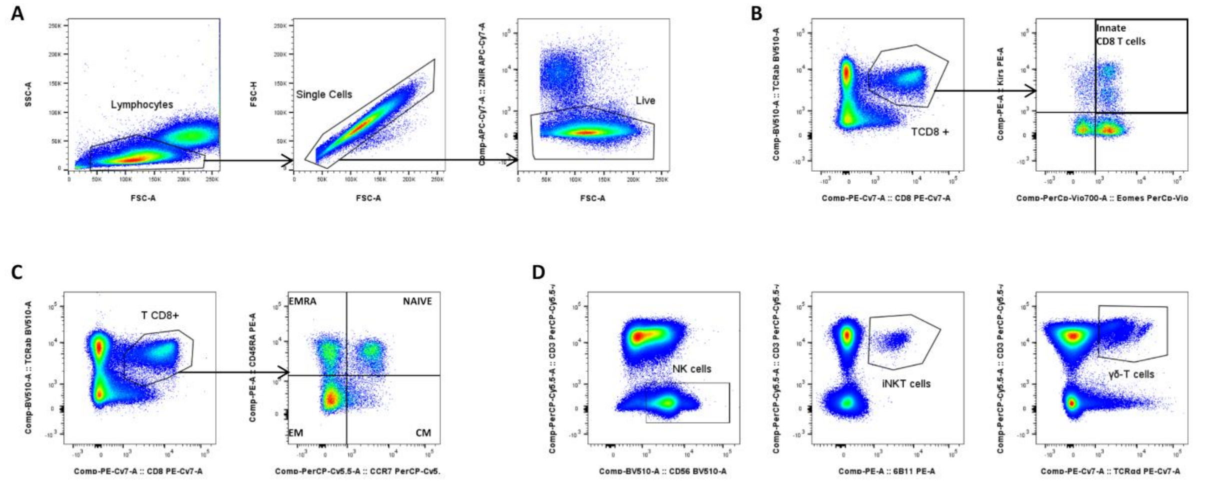

Gating strategies for the identification of innate and conventional T-cells, NK cells, iNKT cells and γδ T-cells. (A) Common gating tree to define live lymphocytes. **(B)** Gating tree for innate CD8 T-cells defined as TCR-αβ(+)CD8(+)KIR/NKG2A(+)Eomes (+) cells. **(C)** Gating tree for memory phenotype of CD8 T-cells defined as TCR-αβ(+)CD8(+) cells, and based on the expression of CD45RA and/or CCR7. **(D)** NK cells, iNKT cells and γδ T-cells are defined as CD3(-)CD56(+) cells, CD3(+)TCR-Vα24-Jα18(+) cells, and CD3(+)TCRpan-γδ(+) cells, respectively.

Supplementary Figure 2

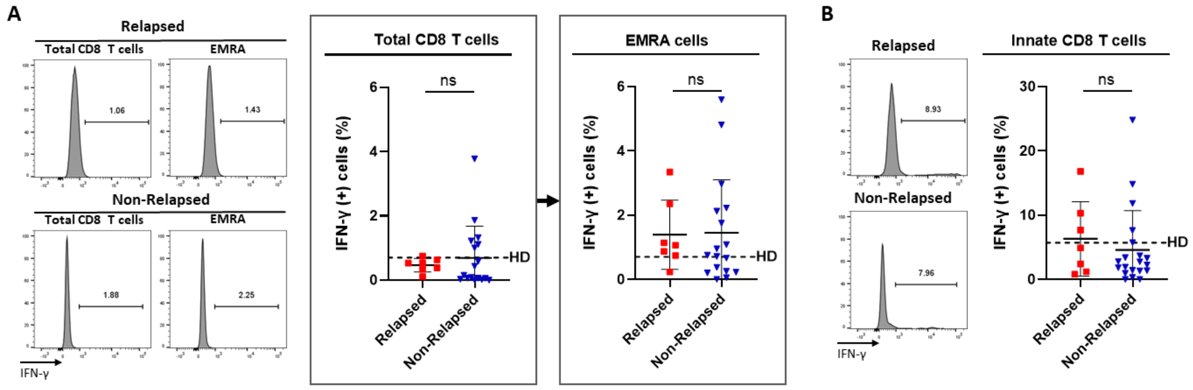

Frequencies of IFN-γ-expressing cells among circulating CD8 T-cell subtypes after innate stimulation: comparison between relapsed and non-relapsed patients at the moment of TKI discontinuation. PBMC were cultured with IL-12+IL-18 for 48 h. **(A)** Frequency of IFN-γ-expressing cells after gating on total CD8 T-cells and EMRA cells, defined as TCR-αβ(+)CD8(+) cells and TCR-αβ(+)CD8(+)CCR7(-)CD45RA(+), respectively, from relapsed (n=7) and non-relapsed (n=17) patients. HD means (dotted lines): 0.7% ± 0.76% of total CD8 T cells; 0.7% ± 0.46% of EMRA cells (n=13). **(B)** Frequency of IFNγ-expressing cells after gating on innate CD8 T-cells, defined as TCR-αβ(+)CD8(+)Eomes(+)KIR/NKG2A(+) cells, from relapsed (n=7) and non-relapsed (n=20) patients. HD mean (dotted line): 5.7% ± 4.9% (n=13). Representative flow cytometry histograms represent expression of IFN-γ in the different cell populations of interest in relapsed (top left) and non-relapsed (bottom left) patients. Numbers in flow cytometry histograms indicate the frequencies of IFN-γ-expressing cells in the indicated cell population. Histograms represent cohort analysis of frequencies of cells expressing IFN-γ (mean ± SD). Each dot represents a relapsed (red squares) or a non-relapsed (blue triangles) patient. Statistical significance was determined by the two-tailed Mann-Whitney non-parametric test. ns: not significant.

Supplementary Figure 3

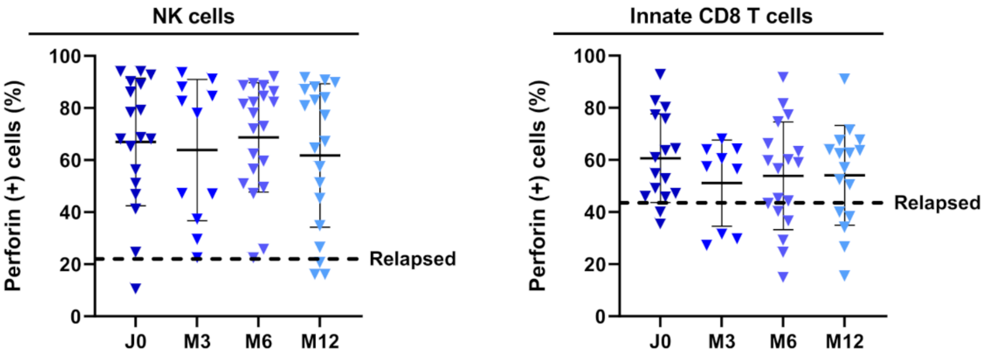

Comparison of frequencies of perforin-expressing NK cell and innate CD8 T-cells between relapsed and non-relapsed patients: longitudinal analysis after treatment discontinuation. Frequencies of perforin-expressing NK cells (left panel) and innate CD8 T-cells (right panel), defined as CD3(-)CD56(+) cells and TCR-αβ(+)CD8(+)Eomes(+)KIR/NKG2A(+) cells, respectively, from non-relapsed patients at the moment of TKI discontinuation (n=18), and 3 months (n=11), 6 months (n=20) and 12 months (n=19) after TKI discontinuation. Relapsed patient means at the moment of TKI cessation (dotted lines): 22% ± 7.1% of NK cells, 43.5% ± 9.1% of innate CD8 T cells (n=4). Of note, values of non-relapsed patients remained higher after 3, 6 and 12 months (p-values for NK cells: 0.0264, 0.0048, 0.0645, respectively; p-values for innate CD8 T-cells: 0.2523, 0.1409, 0.1353, respectively) after TKI cessation, when compared with relapsed patients at the moment of TKI cessation.
Histograms represent kinetics cohort analysis of frequencies of cells expressing perforin (mean ± SD). Each dot represents a non-relapsed patient at the moment of TKI discontinuation, and 3 months, 6 months or 12 months after TKI discontinuation

Supplementary Figure 4

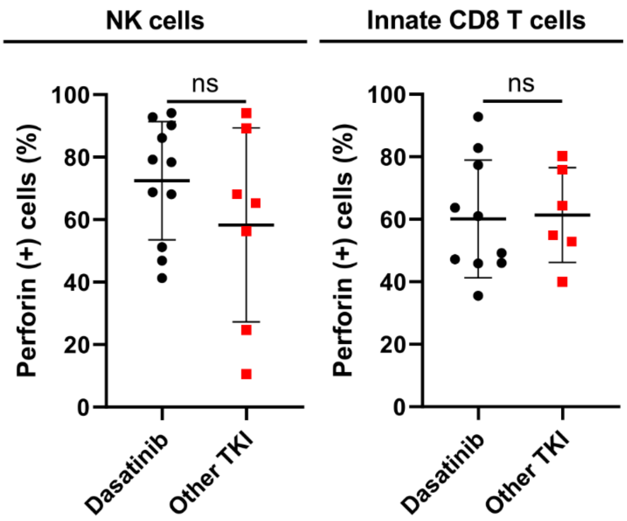

Frequencies of perforin-expressing NK and innate CD8 T-cells from non-relapsed patients: comparison between dasatinib- and other TKI- treated patients. Frequencies of perforin-expressing NK cells (left panel) and innate CD8 T-cells (right panel), defined as CD3(-)CD56(+) cells and TCR-αβ(+)CD8(+)Eomes(+)KIR/NKG2A(+) cells, respectively, from non-relapsed patients treated with dasatanib (n=10) or with other TKI (n=6). Histograms represent cohort analysis of frequencies of cells expressing perforin (mean ± SD) among the indicated cell population. Each dot represents a non-relapsed patient treated with dasatinib (black dots) or a non-relapsed patient treated with other TKI than dasatinib (red squares) at the moment of TKI cessation. Statistical significance was determined by the two-tailed Mann-Whitney non-parametric test. P- value: ns: not significant.

Supplementary Figure 5

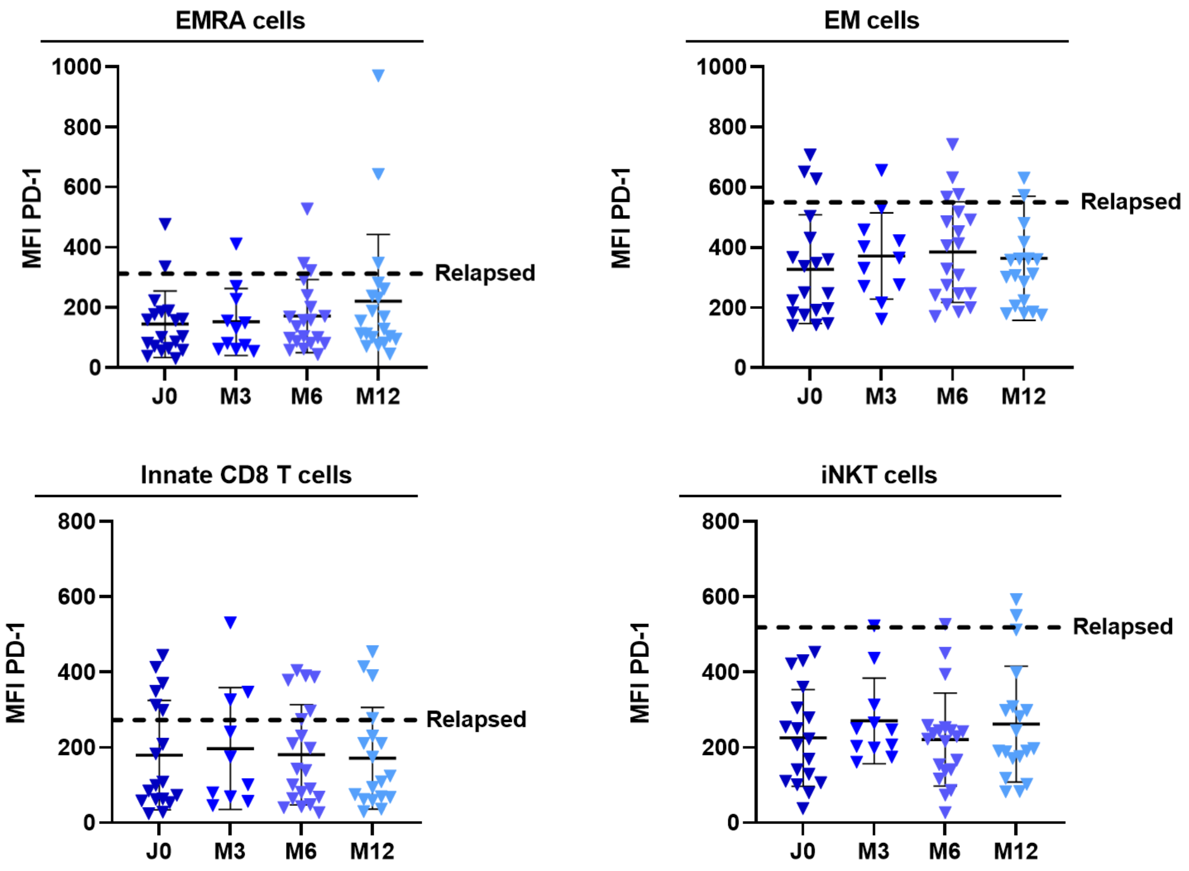

Comparison of frequencies of PD-1 MFI on effector T-cell subsets between relapsed and non-relapsed patients: longitudinal analysis after treatment discontinuation. PD-1 MFI on EMRA cells (upper left panel), defined as TCR-αβ(+)CD8(+)CCR7(-)CD45RA(+), EM cells, (upper right panel) defined as TCR-αβ(+)CD8(+)CCR7(-)CD45RA(-) cells, innate CD8 T-cells (lower left panel), defined as TCR-αβ(+)CD8(+)Eomes(+)KIR/NKG2A(+) cells, and iNKT cells, (lower right panel) defined as CD3(+)TCR-Vα24-Jα18(+) cells, from non-relapsed patients at the moment of TKI discontinuation (n=18), and 3 months (n= 11), 6 months (n=20) and 12 months (n=19) after TKI discontinuation. Relapsed patient means at the moment of TKI cessation (dotted lines): 313 ± 143, 550 ± 153.3, 219 ± 75 and 273 ± 189.6 for EMRA cells, EM cells, iNKT cells, and innate CD8-T cells, respectively (n=5). Of note, values of non-relapsed patients remained lower 3, 6 and 12 months after TKI discontinuation for EM cells (p-values: 0.0346, 0.0549, 0.0056, respectively), EMRA cells (p-values: 0.0346, 0.0263, 0.0716, respectively), and iNKT cells (p-values: 0.0094, 0.0011, 0.0144), and 6 and 12 months after discontinuation for innate CD8-T cells (p-values: 0.0813, 0.0392, respectively), when compared with relapsed patients at the moment of TKI cessation.

Histograms represent kinetics cohort analysis of PD-1 MFI (mean ± SD). Each dot represents a non-relapsed patient at the moment of TKI discontinuation, and 3 months, 6 months or 12 months after TKI discontinuation.

<u>Supplementary Figure 6</u>

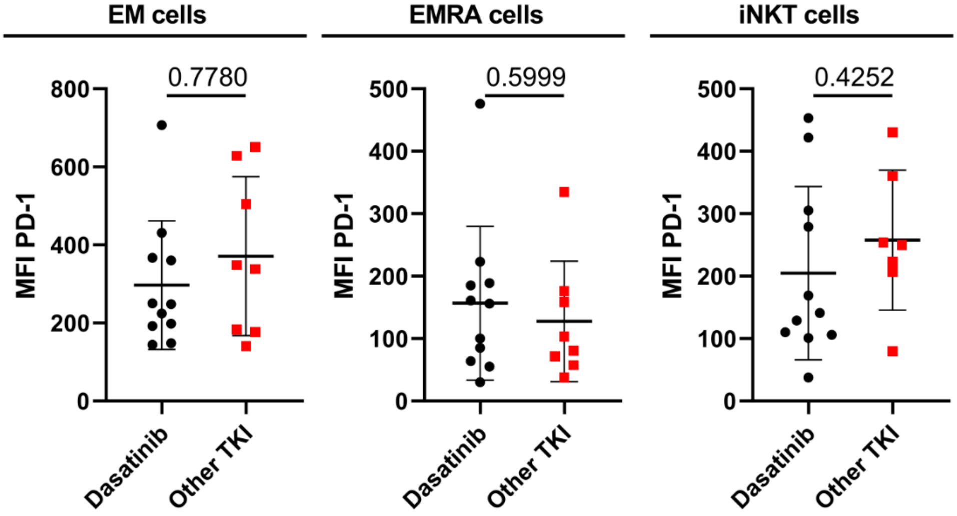

Frequencies of PD-1 MFI on effector T-cell subsets from non-relapsed patients: comparison between dasatinib- and other TKI- treated patients. PD1 MFI on EMRA cells (left panel), defined as TCR-αβ(+)CD8(+)CCR7(-)CD45RA(+), EM cells (medium panel), defined as TCR-αβ(+)CD8(+)CCR7(-)CD45RA(-), and iNKT cells (right panel), defined as CD3(+)TCR-Vα24-Jα18(+), from non-relapsed patients treated with dasatanib (n=11) or with other TKI (n=7). Histograms represent cohort analysis of PD-1 MFI (mean ± SD) among the indicated cell population. Each dot represents a non-relapsed patient treated with dasatinib (black dots) or a non-relapsed patient treated with other TKI than dasatinib (red squares) at the moment of TKI cessation. Statistical significance was determined by the two-tailed Mann-Whitney non-parametric test. P-value: ns: not significant.
